# *Immuno-*μSARS2 Chip: A Peptide-Based Microarray to Assess COVID-19 Prognosis Based on Immunological Fingerprints

**DOI:** 10.1021/acsptsci.4c00727

**Published:** 2025-02-21

**Authors:** Julian Guercetti, Marc Alorda, Luciano Sappia, Roger Galve, Macarena Duran-Corbera, Daniel Pulido, Ginevra Berardi, Miriam Royo, Alicia Lacoma, José Muñoz, Eduardo Padilla, Silvia Castañeda, Elena Sendra, Juan P. Horcajada, Agustín Gutierrez-Galvez, Santiago Marco, J.-Pablo Salvador, M.-Pilar Marco

**Affiliations:** † Nanobiotechnology for Diagnostics Group, Instituto de Química Avanzada de Cataluña, IQAC−CSIC, C/Jordi Girona 18-26, 08034 Barcelona, Spain; ‡ Multivalent Systems for Nanomedicine (MS4N), Instituto de Química Avanzada de Cataluña, IQAC−CSIC, C/Jordi Girona 18-26, 08034 Barcelona, Spain; § CIBER de Bioingeniería, Biomateriales y Nanomedicina (CIBER-BBN), Instituto de Salud Carlos III, 28029 Madrid, Spain; ∥ Servei de Microbiologia, Hospital Universitari Germans Trias i Pujol, Institut Germans Trias i Pujol, 08916 Badalona, Spain; ⊥ CIBER de Enfermedades Respiratorias (CIBERES), Instituto de Salud Carlos III, 28029 Madrid, Spain; # Servicio de Microbiología del Laboratorio de Referencia de Catalunya, 08820 Barcelona, Spain; 7 Servicio de Enfermedades Infecciosas del Hospital del Mar de Barcelona, COVID-MAR group, 08003 Barcelona, Spain; 8 CIBER de Enfermedades Infecciosas (CIBERINFEC), Instituto de Salud Carlos III, 28029 Madrid, Spain; 9 Institute for Bioengineering of Catalonia (IBEC), The Barcelona Institute of Science and Technology, Baldiri Reixac 10-12, 08028 Barcelona, Spain; 10 Department of Electronics and Biomedical Engineering, University of Barcelona, Marti i Franqués 1-11, 08028 Barcelona, Spain

**Keywords:** Microarray, High-throughput, Serological signature, Peptide epitopes, Multiplexation, Machine learning, Clinical diagnostic, Severity prediction, SARS-CoV-2

## Abstract

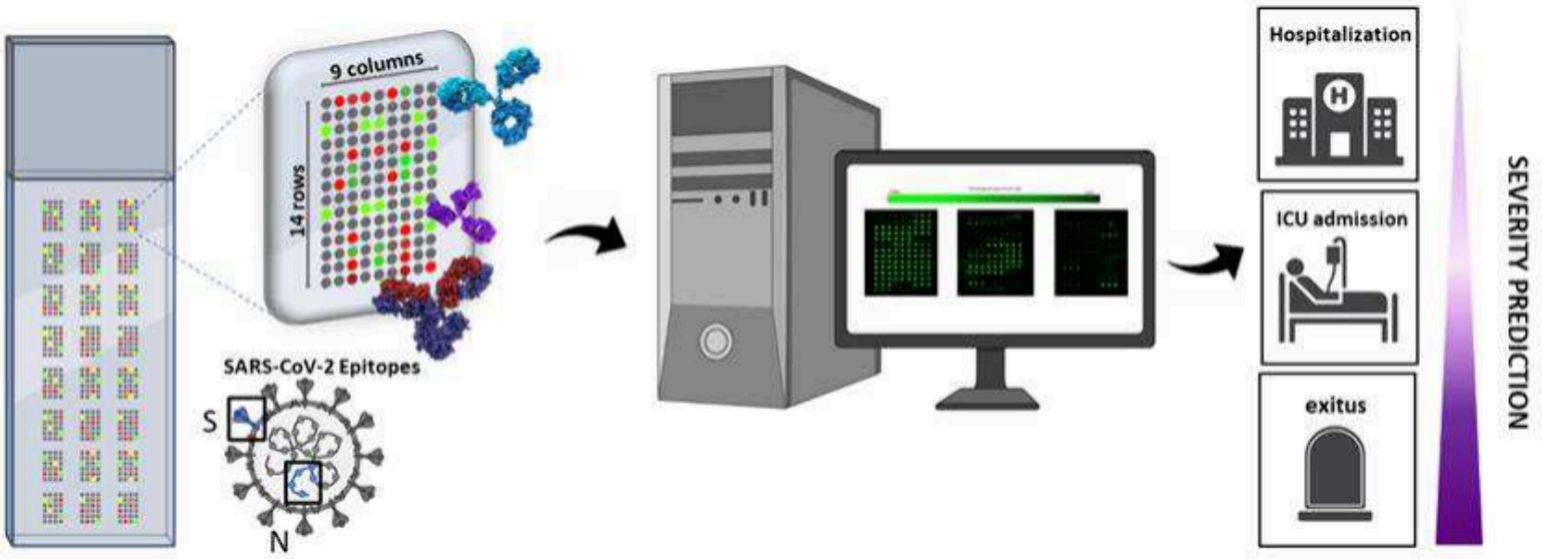

A multiplexed microarray chip (*Immuno*-μSARS2) aiming at providing information on the prognosis of the COVID-19 has been developed. The diagnostic technology records information related to the profile of the immunological response of patients infected by the SARS-CoV-2 virus. The diagnostic technology delivers information on the avidity of the sera against 28 different peptide epitopes and 7 proteins printed on a 25 mm^2^ area of a glass slide. The peptide epitopes (12–15 mer) derived from structural proteins (Spike and Nucleocapsid) have been rationally designed, synthesized, and used to develop *Immuno*-μSARS2 as a multiplexed and high-throughput fluorescent microarray platform. The analysis of 755 human serum samples (321 from PCR+ patients; 288 from PCR– patients; 115 from prepandemic individuals and classified as hospitalized, admitted to intensive-care unit (ICU), and *exitus*) from three independent cohorts has shown that the chips perform with a 98% specificity and 91% sensitivity identifying RT-PCR+ patients. Computational analysis utilized to correlate the immunological signatures of the samples analyzed indicate significant prediction rates against *exitus* conditions with 82% accuracy, ICU admissions with 80% accuracy, and 73% accuracy over hospitalization requirement compared to asymptomatic patients’ fingerprints. The miniaturized microarray chip allows simultaneous determination of 96 samples (24 samples/slide) in 90 min and requires only 10 μL of sera. The diagnostic approach presented for the first time here could have a great value in assisting clinicians in decision-making based on the information provided by the *Immuno*-μSARS2 regarding progression of the disease and could be easily implemented in diagnostics of other infectious diseases.

SARS-CoV-2 (Severe Acute Respiratory Syndrome Coronavirus 2) was identified as the causative agent of coronavirus disease 2019 (COVID-19) and in terms of months spread worldwide, leading to a pandemic.^1−3^ This enveloped positive-sense RNA virus carries a 29 kb genome that encodes four major structural proteins, Spike (S), Envelope (E), Membrane (M), and Nucleocapsid (N), with eight additional accessory proteins and 15 nonstructural components.^4,5^ The S protein is highly expressed on the viral surface, where it is anchored to the membrane and mediates cell entry.^6^ Naturally found as a homotrimer (180 kDa each), it is composed of 1273 aa, characterized by a signal peptide (from aa 1–13) located at the N-terminal domain (NTD) followed by an S1 subunit (from aa 14–685) that contains the receptor binding domain (RBD), allowing specific interaction with the Angiotensin-converting enzyme 2 (ACE2) receptor in the host cells.^7,8^ Finally, the S2 subunit (from 686–1273 aa) corresponds to the transmembrane domain, which is essential for membrane fusion with the host cell.^9^ On the other hand, the N protein (46 kDa, 419 aa) is another essential structural component that is tightly bound to the viral RNA.^10^ The key functions behind the N protein include RNA packaging, replication and assembly, and the formation of ribonucleoprotein complexes to protect it from degradation,^11^ sharing more than 90% of homology with SARS-CoV.^12^

From a clinical perspective, COVID-19 has been characterized by a variety of patient-dependent symptoms, mostly ranging from asymptomatic or mild flu-like symptoms to severe bilateral pneumonia resulting in acute respiratory distress and even death.^13,14^ In addition, fatal to severe cases are mostly found in the elderly population considering age as a risk factor.^15−17^ Disease severity was also correlated with gender-based differences and comorbidities.^18−21^ Another group of patients, who may experience chronic fatigue or pain as well as physiological and neurological symptoms several months postinfection, are diagnosed with long COVID-19.^22^ Reinfection with pre-existent viral exposure or vaccination was also demonstrated using current serological testing.^23^

During the initial phase of the pandemic, the high rate of asymptomatic patients sets a challenge for conventional diagnostics^24,25^ required for faster, user-friendly, and cost-effective alternatives to cover the mass scale testing needs and decentralize diagnostics from laboratories.^26−29^ Following the publication of the virus sequence, PCR based methods were rapidly defined as the gold standard techniques to detect viral presence,^30,31^ requiring trained personnel and a minimum of 2–3 h to obtain results.^32^ However, as the infection spread, the PCR testing facilities collapsed, and access to the necessary reagents became difficult. Antigen tests (known as rapid tests) arose as easier to use alternatives for screening purposes.^26,33,34^ In parallel, serological tests measuring the levels of immunoglobulins (mainly IgG or IgM) rapidly appeared during the first stages of the pandemic, although their diagnostic value was low since they gave positive responses only after one- or two-weeks postinfection.^35^ However, serological tests were useful in epidemiological studies to assess the level of exposure of the population. Serologic point of care (PoC) devices typically detect the level of immunoglobulins against structural proteins such as S and N by immobilizing them in cost-effective and simple configurations^36,37^ like lateral flow immunoassays (LFIAs),^38,39^ Enzyme Linked Immunosorbent Assay (ELISA),^40,41^ and electrochemical devices that show potential as rapid detection tools.^42,43^ Despite the important role of diagnostics in the COVID-19 pandemic, each of these technologies sheds light on the diversity of symptoms and their spread.

Nowadays, it is widely accepted that the host immune response plays a key role in disease control and clinical progression.^44,45^ Adaptive immunity, mediated by B cells, is responsible for the production of specific immunoglobulins (IgA’s, IgM’s, and IgG’s) to target the virus and induce its elimination. According to data, IgG levels showed better correlation with disease progression than other isotypes.^46,47^ Particular interest has been focused toward determination of neutralizing antibodies (NAb’s) due to the natural ability to bind to RBD interfering with the entrance of the virus to the cell.^48,49^ Fluorescent microarrays have been proposed to determine the immune response due to their high sample throughput and multiplexing capabilities generating a huge amount of data.^50−52^ Hence, the serological profile against the complete viral proteome has been assessed at amino acid resolution,^35^ and the stronger immune response has been found to be produced against the N and S1 subunits, the more abundant viral proteins.^53^ In another study, antibodies from 2500 human serum samples were measured against S protein peptides, pointing to the higher diagnostic value of the RBD region.^17^ In parallel, recent reports emphasize the need to develop biostatistical tools and machine learning models to define personalized profiles associated with clinical outcomes.^54,55^

In this scenario, our work has been focused on the rational definition of a discrete panel of peptide epitopes and proteins to identify specific IgG signatures in serum samples that could provide information about the disease progression and prognosis, as a tool to assist clinicians in decision-making. In addition, the analysis of such data also shows the possibility of defining a smaller panel of epitopes to provide data sets that are easier to interpret and suitable for PoC, while lowering the cost of diagnosis. Aiming to favor the implementation and regular use in the clinical field, the need to develop appropriate algorithms to support the interpretation of the molecular signatures obtained has been raised.

## Materials and Methods

### General Methods, Reagents, and Instruments

#### Instruments

The pH and the conductivity of all buffers and solutions were measured with a SevenCompact Duo S213 pH meter (MettlerToledo, Spain). A matrix-assisted laser desorption ionization time-of-flight mass spectrometry (MALDI-TOF MS) Bruker Autoflex III Smartbeam spectrometer (Billerica, Massachusetts) was used to determine the peptide densities of the BSA bioconjugates. Probe deposition was performed using an automated spotter piezo-driven sciFLEXMICROARRAYER S3-Scienion AG spotter (Scienion AG, Berlin, Germany). Fluorescent signal acquisition was possible through a dual color microarray scanner InnoScan 710 (Innopsys, Carbonne, France) at wavelengths 555 and 647 nm. GraphPad Prism V7.0 (GraphPad Software Inc., San Diego, USA) was used to plot the data obtained from the analysis.

#### Buffers

The buffer was 0.01 M phosphate buffer (1.48 mM KH_2_PO_4_ and 8.3 mM Na_2_HPO_4_) in 0.8% saline solution (137 mmol·L^–1^ NaCl, 2.7 mmol·L^–1^ KCl) at pH 7.5. PBST is the phosphate buffer solution (PBS) previously described with 0.05% Tween 20. The printing buffer was PBS 10 mM (filtered 0.2 μm). The sample dilution buffer consisted of 10 mM PBST with 0.5% (w/v) bovine serum albumin (BSA, Merck KGaA, Germany).

#### Immunoreagents

Normal human serum from Merck KGaA (Darmstadt, Germany) collected from the clot os healthy donors was used as a negative control. Secondary labeled antibodies: Goat Anti-Rabbit IgM mu chain (Alexa Fluor 647) and Goat Anti-Rabbit IgG H&L (Alexa Fluor 555) were acquired from Abcam plc. (Cambridge, United Kingdom). Rabbit anti-Human TRITC IgG (ref ab6756) and Rabbit anti-Human Alexa fluor 647 IgM conjugate (ref ab150191) were also purchased from Abcam plc. (Cambridge, United Kingdom). The rest of the reagents printed in the microarray such as viral recombinant proteins are detailed in Table S2. On the other hand, two in-house produced reference antisera (As410 and As414) were generated after three immunizations in rabbits with the recombinant portion of S1 and NC for As410 and As414, respectively. The corresponding antisera were purified using an AKTA system equipped with a protein G column, yielding the polyclonal fractions defined as PAb410 and PAb414. To optimize of the array concentrations, the antisera As410 (S1) and As414 (N) were utilized as positive controls (CTR+As S1 and N) and the purified polyclonal antibodies CTR+PAb S1 and CTR+PAb N were then used as reagents for quality control of the print. Preimmune serums from rabbits As410 and As414 were used as standard negative serum (CTR-As S1 and N).

### Rational Peptide Design

A total of 22 selected peptide sequences (plus 6 sequences with mutations) from SARS-CoV-2 were designed (6 from the N protein and 16 from the S protein). Several databases including PubMed, NCBI, and UniProt were used in addition to BLAST for sequence alignment. Additionally, the B cell epitope predictor software BepiPred-2.0 was implemented, defining an epitope-threshold of 0.6.^56^ In addition, Figure S1 and Table S1 show the final exact peptide sequences selected in concordance with the discussion carried out in this article.

### Peptide Synthesis and BSA Conjugation

Peptides **P1** to **P10** were synthesized manually following the standard Fmoc/*t*Bu solid-phase synthesis strategy. Peptides **P11** to **P22** were synthesized using the standard Fmoc/*t*Bu solid-phase synthesis strategy in an automated microwave-assisted peptide synthesizer (see Supporting Information (SI) for details of the peptide synthesis). The peptides ranged in length from 12 to 15 amino acids and were synthesized with a cysteine (Cys) at either N- or C-terminus for orthogonal chemical bioconjugation to BSA using N-succinimidyl 3-maleimidopropionate (N-SMP) cross-linker.^57^ All the bioconjugates were purified by dialysis and characterized by matrix-assisted laser desorption ionization time-of-flight mass spectrometry (MALDI-TOF/MS) yielding an average density of 5 to 7 peptides per BSA molecule (See SI document).

### Fluorescent Microarray

#### Microarray Printing

Microscope slides (plain precleaned 75 × 25 mm) purchased from Corning Inc. (Corning, NY, USA) were silanized with GPTMS (3-glycidoxypropyltrimethoxysilane) according to described procedures.^58^ The recombinant proteins (N-SARS, N-SARS2, S1-SARS, S1-SARS2, S1-Trimer-SARS2, S1+S2, and RBD) and the peptide epitopes conjugated to BSA (P_1–22_-BSA) were spotted onto the activated slides in the form of BSA bioconjugates distributed in a 9 × 14 matrix on each microarray chip. Optimized spotting concentrations were defined by evaluating the binding of serial dilutions (1/1600; 1/3200; 1/6400 in PBST) of the CTR+As S and CTR+As N on microarray chips spotted with serial dilutions of the P_1–22_-BSA bioconjugates (1 and 0.5 mg mL^–1^ in printing buffer) or the proteins (at 100, 50, and 25 μg mL^–1^ in printing buffer). Suitable results were obtained in most cases by using a 1/6400 dilution for the CTR+As and 0.5 mg mL^–1^ and 25 μg·mL^–1^ for printing P_1–22_-BSA and the proteins, respectively.

The secondary labeled antibodies utilized in the immunoassay (Rabbit anti-Human TRITC IgG and Rabbit anti-Human Alexa fluor 647 IgM) were spotted on the array, as internal controls for fluorescence performance or reagents. Table S2 provides information on the final concentrations used for the microarray (Figure S3) showing the matrix distribution. For the reagent depositions, a piezo dispense capillary (PDC) 70 type 4 (voltage 86 V and pulse width 49 μs) was employed. Optimization studies were performed to ensure enough signal and suitable spot definition which was achieved by depositing 2 consecutive drops of 350 pL per spot (25 °C and 60% humidity), drying for 1 h at RT, and then kept at 4 °C until use for a maximum of 5 days (see Figure S4). Over each 75 × 25 mm glass slide, 24 independent microarray chips were printed. Each chip contained 35 epitopes in three replicate spots. The matrix size was adjusted to fit with the dimensions of the ArrayIt holder (ArrayIt Corp, Sunnyvale, CA, USA) used for simultaneous sample analysis.

#### Microarray Protocol

Four printed glass slides were inserted in the ArrayIt gasket creating a 96-well microplate like configuration. The assay protocol started by washing the slides (PBST, 200 μL/well) and then adding the human serum samples (10 μL of serum diluted 1/5 with PBST containing 0.5% BSA) and incubating them for 60 min at RT. The slides were washed again (PBST, 3 × 200 μL/well), and a mixture of secondary TRITC and Alexa fluor 647 labeled antihuman IgG (1/500 in PBST) and IgM (1/250 in PBST), respectively, was added (200 μL/well) and incubated for 30 min at RT protected from the light. Finally, the slides were washed (PBST, 3 × 200 μL/well, plus 1× Milli Q water 200 μL/well) and dried with a N_2_ stream before signal acquisition with the microarray scanner at 555 nm (for IgG) and 647 nm (for IgM) simultaneously using the Mapix Analysis software V.7.4.0 integrated under the following conditions: 555 nm channel (green, IgG), Gain = 3, Power Low 5 mW; 647 channel (red, IgM) Gain = 10, Power Low 5 mW. The fluorescence recorded on each epitope spot when measuring blank (negative for SARS-Cov2) serum corresponding to nonspecific adsorptions was subtracted from the values recorded with the unknown positive samples.

For quality controls, matrix deposition quality assessment was performed for each printed batch by running one slide (out of 20 slides) with CTR+PAb S and CTR+PAb N to monitor printing batch variability between different sets of slides. Those batches with less than 20% of variability in printing response were selected for the analysis of clinical samples (see interday variability achieved in Figure S4).

### Serum Samples

A total of 755 human serum samples from three independent patient cohorts were included in this study: Cohort 1, Aragon Health System Biobank; Cohort 2, Germans Trias i Pujol University Hospital (HUGTP); Cohort 3: MarBiobanc (Parc Salut Mar). RT-PCR positive (POS) or negative (NEG) was used as the primary classification criteria. The sampling period ranged from 01/05/2020 to 01/08/2020 assuming the prevalence of the Wuhan variant and the lack of available vaccines. Clinical information related to disease progression and outcome (nonspecified, asymptomatic/mild symptoms, hospitalization, intensive-care unit (ICU), and *exitus*) was provided by the different institutions and handled according to the recommendations of the Clinical Trials Regulation (CTR or Regulation (EU) No 536/2014 of the European Parliament and of The Council of 16 April 2014 on clinical trials) regarding confidentiality and anonymization of the data. The experimental procedures and research objectives were approved by the corresponding Ethics and Scientific Committees of the healthcare and research institutions.

All samples were measured, but for the biostatistical analysis, only those obtained 10 days after symptom onset were considered.^35^ As a negative control group, prepandemic samples obtained from healthy donors during the first months of year 2019 were used. Internal quality controls were included on each glass slide by designating two wells (out of the 24 available) to analyze a negative human serum sample (10 μL). Table 1 shows a summary of the samples analyzed and the cohort distribution according to the RT-PCR results and prepandemic samples (left side). In addition, the right side of Table 1 shows a reduced selection of samples classified according to the clinical outcome.

**Table 1:** Description of the Clinical Samples According to Their Origin, RT-PCR Classification, and Clinical Outcome^a^

	Validation			Clinical Outcome				
Cohort	RT-PCR+	RT-PCR–	Prepandemic	Hospitalization	ICU	*Exitus*	Asymptomatic/mild	Nonspecified
1	143	178		55	24	37	25	2
2	81	91	18	17	6	2	37	19
3	96	51	97	47	28	9	1	11

a Three independent patient cohorts were included in this study: Cohort 1, Aragon Health System Biobank; Cohort 2: Germans Trias I Pujol University Hospital (HUGTP); Cohort 3: MarBiobanc (Parc Salut Mar). Samples classified according to clinical outcomes were selected as independent subgroups from RT-PCR+ samples but do not strictly represent the total number of RT-PCR+ samples from each cohort.

### Biostatistical Analysis

The signal from each epitope was obtained as the average fluorescence intensity of three replicate spots from each sample, subtracting the average fluorescence obtained with the control human serum measured in each slide and afterward and then applying a logarithmic scale. The data were normalized within each cohort, taking 100% of the highest value measured in relative fluorescence units and 0% to 0 relative fluorescence units’ value of the control human serum samples used.

Initial sample classification with respect to the gold standard RT-PCR technique to diagnose infection was estimated using different data analysis models consisting of multivariate approaches. For the multivariate model, classification and regression models were generated in order to assess the discrimination power of the microarray for RT-PCR+ vs prepandemic samples and separately define severity predictions. Additionally, feature selection techniques were applied to find a reduced number of epitopes with a significant predictive performance. Principal component analysis was used throughout the data evaluation for visualization purposes. For classification and regression, partial least-squares (PLS), random forest (RF), and K-nearest neighbors (KNN) were utilized. Random forest provided the best performance among all. Variable importance for decision trees in a random forest was determined with the VImp function. Double cross-validation with a 10-fold cross-validation was implemented in this analysis. Epitope selection was performed using feed forward floating feature selection and genetic algorithms as search strategies following a wrapper approach based on KNN and random forest as the criterion function.

## Results and Discussion

Despite the extensive research work that has been done to understand the nature of the SARS-CoV2 virus infection and host interaction, many questions still arise regarding the different symptomatology and disease progression of the disease. While some people were asymptomatic, others experienced conditions that may fluctuate from mild symptoms (fever, chills, sore throat), moderate symptoms (muscle aches, fatigue, persistent cough, appetite loss, change of taste, shortness of breath, etc.), severe symptoms (difficulty breathing, confusion, persistent pain or chest pressure, etc.), or even worse prognosis requiring immediate medical attention or hospitalization. Certain patients experienced a hyperinflammatory state secondary to the excessive production of cytokines (severe systemic inflammatory syndrome, SIRS, cytokine storm), which resulted in a drastic self-reinforcement of various feedback mechanisms, which ultimately led to systemic damage, multiorgan failure, or death. The etiology of such diversity is still not completely understood, but it seems clear that the response of the immune system seems to be decisive for the outcome of infection.^19,59^ The aforementioned scenario prompted us to attempt to develop a diagnostic technology to profile the adaptive immune response of the patients, aiming to provide insights that could help understand the variety of individual disease patterns or even predict the evolution of the infection in each case. For this purpose, we proposed the development of a peptide epitope-fluorescence microarray chip aimed at investigating the variety of immunological responses toward the virus. Hence, this required the identification of peptide epitopes from two of the most relevant structural proteins of SARS-CoV-2, identified as S and N.

A high-throughput diagnostic tool was envisaged with multiple microarray chips, each containing a panel of peptide epitopes and proteins from the virus, with the objective to investigate a potential correlation between the profiles of the patient immunological responses with respect to the symptomatology and disease progression. In addition, we aimed to identify and select a set of epitopes showing a robust predictive value toward clinical severity based on machine learning techniques, with the final aim to facilitate clinical implementation of a more cost-efficient and easier to interpret diagnostic solution without reducing clinical performance and reliability.

### Rational Selection of the Targeted Peptides

In first instance, we addressed the identification of potential peptide epitopes that were selective for SARS-CoV-2 and not for another coronavirus (SARS-CoV and MERS). Hence, the S1 subunit shared 64% of homology among SARS-CoV and SARS-CoV2^60^ and 90.3% for the corresponding N proteins,^61^ making the discrimination of the immunological response toward these related viruses very challenging. Given the high homology of the protein sequences, recombinant S1 and N proteins from both SARS-CoV and SARS-CoV-2 were included in the microarray. It should be noted, however, that as the study progressed, the higher prevalence of the novel coronavirus compared to the pre-existing coronaviruses at that time rendered any attempt to discriminate between the two viruses irrelevant. Post-translational modifications were excluded from the analysis because they would have increased the complexity of the chemical synthesis of the peptides.^62,63^ On the other hand, either the protective or immune evasion role of glycosylation sites was still under discussion.^64,65^

**Figure 1:**
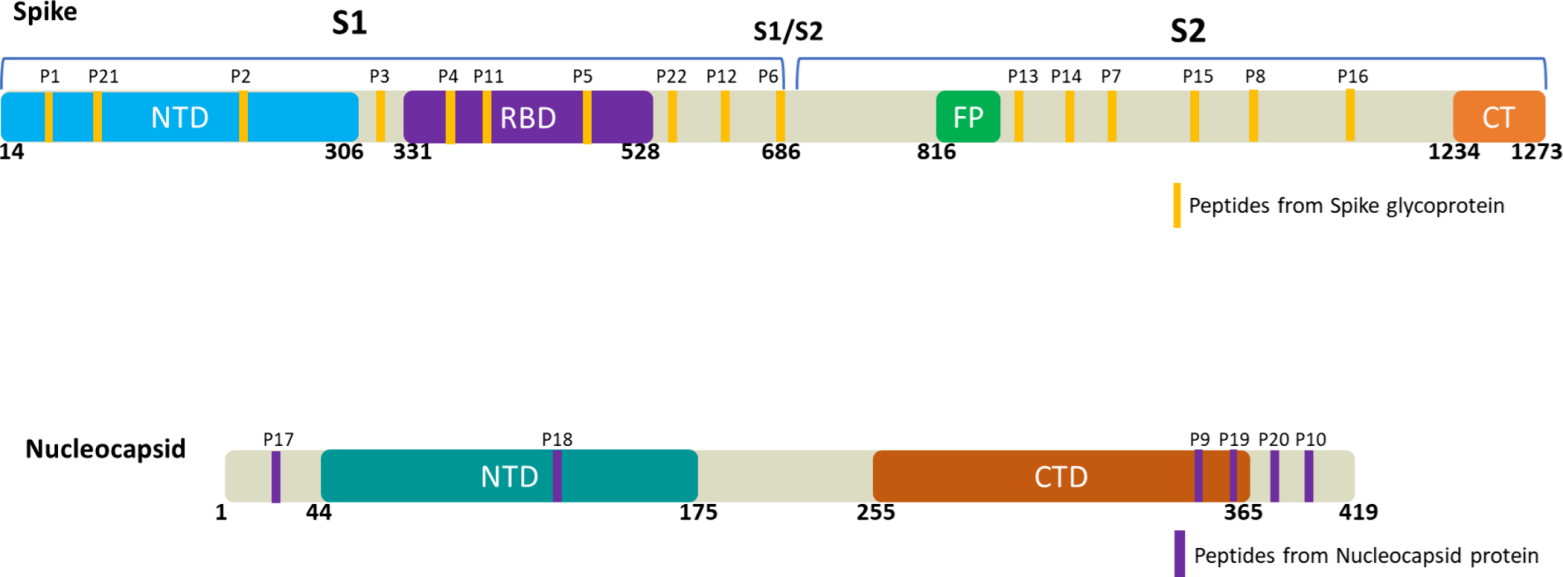
Schematic representation of discrete peptides selected from Spike and Nucleocapsid proteins. A total of 22 linear peptide sequences, in yellow (S) and purple (N), expressed across the original protein (Wuhan Variant) considering functional domains of Spike glycoprotein and Nucleocapsid protein.

Based on literature reports and described computational tools, 28 peptide sequences derived from S and N structural proteins of the SARS-CoV2 virus were rationally selected (Figure 1). Sequence antigenicity, accessibility, avoiding glycosylation sites, length and chemical coupling conditions were some of the criteria used to define the most appropriate peptide sequences. According to the epitope prediction software used (Bepi-Pred2.0), the S1 subunit of the S protein and the C-terminal region of N were highly exposed and consequently more likely to be located between the moieties of the proteins involved in eliciting the immune response. On this basis, several relevant linear peptide epitopes of the S1 region (**P1**, **P21**, **P2**, and **P3**) and the C-terminal region of the N protein (**P9**, **P10**, **P19**, and **P20**) were selected according to the above criteria (Figure S1).

The RBD was carefully studied as the incorporation of such a protein moiety or certain linear peptides would allow targeting of the presence of neutralizing antibodies.^66^ Accordingly, the recombinant RBD protein and four linear peptides (**P4**, **P11**, **P5**, and **P22**) from this region were included in the peptide microarray chip.

At the same time, reported data with respect to the potential immunogenicity of the different regions of the protein were taken into account. For example, Zheng et al.^67^ identified dominant epitopes using surface accessibility and antigenicity scores. On the other hand, *in silico* analysis performed^68^ to define immunogenic sequences from SARS-CoV-2 pointed to five regions, conserved also in SARS, between amino acid residues 274–306, 510–586, 587–628, 784–803, and 870–893. Furthermore, peptide epitopes (**P7**, **P14**, **P15**, and **P16**) from S2 subunit, corresponding to amino acid sequences 776–787, 802–803, 911–920, 982–992, and 1002–1012, were selected based on the prediction models developed by Guevarra and collaborators.^69^

Moreover, reported experimental results that consistently pointed to specific immunogenic domains^70−73^ were also considered for further validation. For instance, the work of Wang et al.^73^ allowed the evaluation of the entire viral proteome using an epitope microarray with amino acid resolution to experimentally determine the best candidates for the recognition of IgM and IgG. Approximately 1000 peptides were immobilized and tested against 10 COVID-19 patients and 10 control patients. As a result, specific sequences located at the S protein including residues 806–820 (**P7**, LPDPSKPSKRSFIED), residues 456–460 (FRKSN), and residues 166–170 (**P18**, TLPKG) of the N protein were validated. Likewise, Li et al.^74^ designed and synthesized a microarray consisting of 211 peptides derived from the S protein to identify potential neutralizing antibodies of 55 sera from convalescent COVID-19 patients. The authors identified three immunodominant regions within the S protein: The C-terminal domain at residues 553–654, the RBD at residues 487–488, and two regions of the S2 domain at residues 764–829 (**P14**) and 1148–1159 (**P16**).

Finally, during the development of this work, new SARS-CoV-2 variants emerged and were characterized to contain specific mutations on the S protein. The relevance of identifying the variants of concern (VoCs) causing the infection was found to enhance the power of the technology. Therefore, with this purpose, as an additional feature, immunogenic peptides from the original Wuhan strain were selected and the exact sequences containing representative mutations from the different VoCs were identified for inclusion on the microarray chip under development. The peptides selected took into account the variants with higher prevalence in Catalonia, owing to the precedence of the samples used to validate this first prototype of the chip: *alpha* (B.1.1.7), *delta* (B.1.617.2),^75^ and *omicron* (B.1.1.529)^76^ as well as others with minor impacts like *beta* (B.1.351) and *gamma* (P.1). Consequently, peptide epitopes of the original and corresponding variants were designed such as **P21**/**P21b** (*alpha* with a deletion in residues ΔH69, ΔH70),^77^**P11**/**P11b** (K417N, derived from the *beta* linage)/**P11c** (K417T, specific for *gamma*) or **P6**/**P6b** (P681H, described in the *alpha* variant)/**P6c** (P681R, found in *delta*). In addition, a peptide **P22b** (N501Y) with a common mutation shared by the *alpha*, *beta*, *gamma*, and *omicron* variants was also proposed in contrast to the original **P22** sequence. The N protein underwent a similar analysis, but no relevant sequences for variants of concern were selected due to the lower frequency of mutations compared to the S protein.

### Synthesis of the Peptides and Bioconjugates

The sequences of the selected peptide epitopes were synthesized by solid-phase synthesis and characterized by spectrometric means (Table S1). All the peptides incorporated an additional Cys residue for bioconjugation, mainly located at the N terminal site, except for **P13** in which the location was at the C-terminal region to favor the exposure of the N domain, which will be accessible after furin cleavage, and for **P5** on which there was already a cysteine in the original sequence. Spotting BSA-peptide bioconjugate microarrays instead of the peptides aimed to accomplish more homogeneous spots by increasing peptide solubility and conferring adequate spatial distribution. The same strategy has been reported in other peptide microarray approaches.^74^

Coupling of the peptides to BSA was performed using an orthogonal chemistry approach that did not interfere with other functional groups present on the remaining amino acid residues. For this purpose, N-SMP (*N*-succinimidyl 3-maleimidopropionate) was used as a heterobifunctional cross-linker that allows the attachment of the amino groups of the free/accessible lysines of BSA to the linker via its NHS-active ester on a first step and the thiol groups from cysteine residues of the peptide through the maleimide functionality by a Michael addition on a second step. According to MALDI-TOF-MS/MS analyses, approximately 15 N-SMP cross-linker moieties were incorporated onto BSA in the first step, while the second bioconjugation step usually rendered between 5 to 7 peptides linked to the BSA molecule (see Table S1). We carefully attempted to obtain similar bioconjugation ratios for all peptides in order to ensure comparable immunochemical responses on the multiplexed microarray chip.

### Microarray Chip Manufacturing

The spotting concentrations for each protein solution were optimized to achieve a comparable fluorescent response using As410 and As414 after performing matrix characterization experiments, evidenced in the QC study in Figure S4. An initial study of the concentration of the protein (20 to 100 μg mL^–1^) and peptide bioconjugate (1 and 0.5 mg mL^–1^) solutions was conducted in separate microarray chips. The lower concentration was chosen to give at least a 10 000 RFU’s response after incubation with control antisera As410 (Anti S1) and As414 (Anti N) at 5 μg mL^–1^ was selected. Subsequently, microarray chips integrating the peptide-BSA conjugates (0.5 mg mL^–1^) and recombinant proteins (20 μg mL^–1^) were manufactured. The positive control antisera (As410 and As414) were used to ensure the reproducibility of microarray production batches. Interday variability evaluated on independent chips printed in different days evidenced a coefficient of variation of less than 20% in the signal at epitope level (see Figure S2), indicating good manufacturing reproducibility and performance. Moreover, each microarray chip contained triplicate spots of BSA (500 μg mL^–1^) to assess potential nonspecific interactions of the antisera with the protein or the peptides bioconjugates (see Figure S3). Negative values on these spots ruled out the possibility that the signal recorded on the spots of the peptide epitopes could be due to an unexpected recognition of the protein rather than the selected peptide sequences. Finally, the microarray chip also contained triplicate spots of human IgG and human IgM microarrays to corroborate the performance of the fluorescent antihuman-IgG and antihuman-IgM.

### Performance of the Microarray Chip

**Figure 2:**
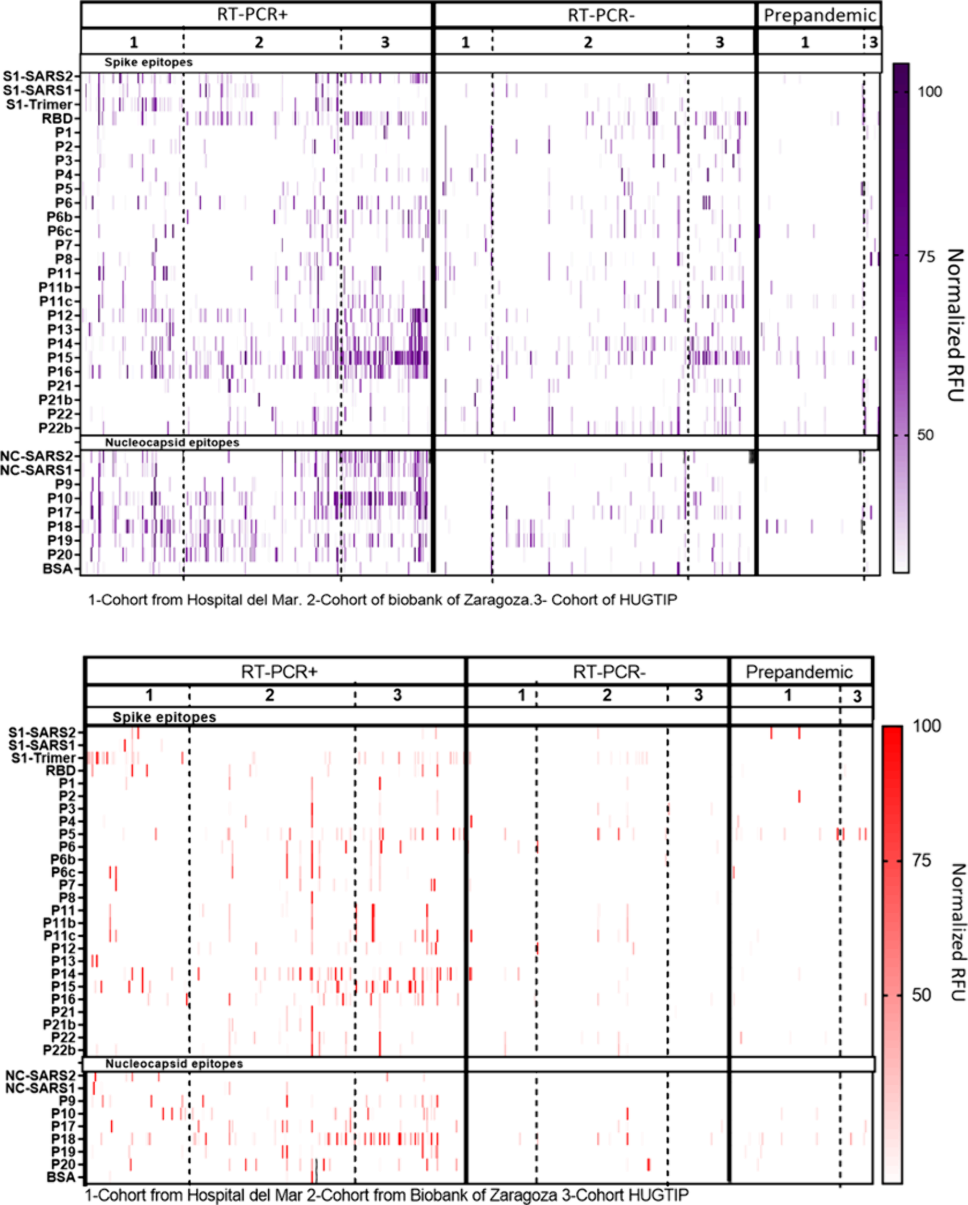
IgG and IgM response heatmap. Landscape of IgG and IgM mediated responses detected from the analysis of 755 human serum samples from three different cohorts of patients, classified according to RT-PCR groups and prepandemic samples as negative controls. The *y*-axis represents individual epitopes constituting the microarray (viral peptides and proteins), while the *x*-axis corresponds to the serum samples from different patients. The intensity scale indicates the signal of IgG and IgM antibodies binding from 0 (white) to 100 (red) in normalized RFU. The data shown corresponds to the average of the signal intensity from three spots for each microarray’s chips. Each slide contained 24 spotted microarrays allowing 22 samples +2 control (commercial serum). The blank signal obtained from the controls was subtracted for each sample.

The analysis of the 755 human antisera samples revealed distinct IgG fingerprints, as shown in Figure 2. The spots with the recombinant proteins and the N derived peptide-BSA bioconjugates showed high fluorescence, as expected and predicted by *in silico* analysis. However, the signal recorded for the S2 derived sequences also indicated strong immunogenicity compared to the peptides of other S protein regions studied, resulting in a promising target for IgG mediated neutralization and serological diagnostic strategies. Although not all the peptides were identified as immunodominant sequences, **P10** together with **P17**, **P18**, and **P19** from the N protein and **P14**, **P15**, and **P16** from S2 showed consistent results across different cohorts with a significant response.

Unfortunately, the immunological profiles potentially generated by the different VoCs could not be evaluated across this work because the cohorts under analysis were mainly affected by Wuhan (see sequences **P6b**, **P6c**, **P11b**, **P11c**, **P21b**, and **P22b** in Figure 2). However, at this stage, the incorporation of these sequences in the microarray chip allowed an assessment of the effect of these mutations on the antibody response. Thus, although a thorough investigation of the effect of these mutations was not carried out, it was possible to observe differences in the intensity of the signals recorded due to these small peptide sequence modifications (see Figure S5). These results therefore suggest that the present chip could be used to diagnose which VoC has caused the infection. The integration of bioinformatic tools could help to evidence such phenomena with further significance (Figure 2), encouraging the performance of further experiments to probe this serological discrimination of VoCs using appropriate patient cohorts and serum samples.

From these analyses, it was decided to continue the studies focusing on the IgG fingerprints that, in general, provided a higher fluorescence intensity (30 000–40 000 RFU’s on average) and well differentiated response patterns regarding RT-PCR positive and prepandemic samples. The intensity of the signals of the IgM profiles was in general lower (around 10 000 to 20 000 RFU’s) with apparently lower discrimination between these two groups. The lower quality of the IgM profiles could be attributed to an inadequate sampling window selection due to the circumstances when these samples were obtained. Hence, the IgM isotype is expressed earlier than IgG’s (around days 3 to 5) and clears much faster. Additionally, the relatively lower abundance of IgM’s in serum compared to IgG’s could lead to lower levels of detectability, considering that a reduced sample volume was required to perform the assay. An alternative approach could contemplate increasing the sample volume used in the assay and delimiting much better the sample collection period to monitor IgM exclusively. These findings are supported by the observations reported by different authors, suggesting the more accurate predictive value of the IgG response in respect to other isotypes.^78^ Nevertheless, the simultaneous monitoring of both immunoglobulins (IgG/IgM) in combination with viral detection techniques would probably provide more precise information on the current immunological status of the patient.

It is also worth noticing the added value of the *Immuno*-μSARS2 chip to identify patients classified as RT-PCR– but with a positive immunological response to the virus (further discussed in the Supporting Information). Hence, a limitation of the RT-PCR is that if the viral load at the time of sample collection is low (initial phase or days after the peak of the infection), then false negatives can be recorded. Thus, the 288 samples defined as RT-PCR– from the cohorts were included in the final analysis, increasing the total number of samples over 700. After the evaluation, the *Immuno*-μSARS2 chip showed a 20–25% rate in terms of immunological response to viral epitopes evaluated.

### Immunological Value of the Peptide Epitopes Selected

Monitoring of the serological status of the population has been fundamental in epidemiological studies, correlating the true prevalence of the infection with the presence of the antibody in the sera of the population. However, in this study, we aimed at demonstrating that the molecular immunological signatures recorded with respect to the recognition of the different epitopes could complement other diagnostic techniques (PCR or antigen test methods), providing additional information regarding the particular individual immunological response to the infection.

As can be observed in Figure 2, the first analyses of the immunological profiles of response revealed a well-defined group of peptides that showed elevated IgG titers and were commonly recognized among the RT-PCR+ samples from the three patient cohorts. Thus, a univariate analysis of the immunological responses toward each peptide epitope (see Figure S6) revealed that, from the 35 epitopes assessed, 30 showed significant positive performance regarding seropositive classification of the RT-PCR+ samples, suggesting that most of them could be used as accurate seroprevalence predictors. However, peptides **P12**, from S1 subunit, **P14**, **P15**, and **P16**, belonging to S2 subunit, and peptides **P10** to **P18**, from N protein, displayed higher fluorescent intensities in comparison to the rest of epitopes included in the matrix.^79^ These results suggested certain immunodominant regions around the S2 mentioned peptide sequences and the C terminal domain of the nucleocapsid.

Regarding the response to the recombinant RBD protein and the peptides derived from the same region included in the matrix to assess the presence of neutralizing antibodies, a much higher signal was observed on the RBD spots with respect to the corresponding peptide sequences selected from the same region (**P4**, **P11**, **P5**, and **P22**), suggesting higher antibody avidity for the epitopes in the native conformation than on the linear form of the synthesized peptides.^80^ Interestingly, the signal recorded on other recombinant protein spots was comparable to that of certain particular peptides included in the matrix, despite the fact that a higher response was expected for the whole proteins as they naturally carry multiple epitopes. However, in light of the results delivered by the RBD peptides and the possibility that the presence of proteins in the same chip could mask the response toward the peptide epitopes, it was decided to spot the recombinant proteins of the virus at lower concentration (an order of magnitude less than peptides).

### Epitope Correlation Analysis

In an attempt to interpret the complex signal responses toward multiple epitopes obtained for each sample on the microarray, Pearson’s correlation studies were performed comparing the signal detected across the chip to define correlated epitopes and identify those providing redundant information. The aim of this approach was to determine whether linear peptides provided comparable detectability to recombinant proteins or whether smaller peptide clusters could preserve the diagnostics, minimizing costs and simplifying signal interpretation. Figure 4 shows a Pearsons’s correlation map where three major clusters can be identified, explaining similar behavior among the peptides or proteins associated. A stronger reddish intensity indicates higher correlation with comparable intensity detected from respective epitopes over the samples under study.

As it can be observed, some peptides seem to share a similar immunological behavior to the structural proteins in terms of the response recorded in the microarray, despite the differences in size, secondary and tertiary structure, and steric constraints. Therefore, the cluster composed of **P16**, **P14**, **P9**, **P19**, **P20**, and NC-SARS shows correlated behavior in respect to the immunological response recorded. Considering the high homology (more than 90%) between N proteins in SARS and SARS2, the correlation of the response of the NC-SARS with the peptides **P9**, **P19**, and **P20** is not surprising. However, the correlation of epitopes **P16** and **P14** from the S2 subunit evidenced common immunoreactive profiles independent of the protein origin. A different cluster was also identified composed of NC-SARS2, **P18**, **P21b**, **P12**, **P17**, **P22**, **P21**, **P22b**, RBD, S1-SARS2, S1-SARS, and **P10**. Once again, this correlation evidences that some proteins like NC-SARS2, RBD, S1-SARS2, and S1-SARS elicit comparable IgG responses to the rationally selected linear peptides belonging to S protein (**P21b**, **P12**, **P22**, **P21**, **P22b**) and N protein (**P18**, **P17**, **P10**). All the peptides derived from the N protein are immunologically correlated with the recombinant proteins spotted on the chip. Particularly, the behavior of **P10** that showed strong correlations with the proteins NC-SARS2, RBD, and S1-SARS2 and related structural N derived peptides like **P17**, **P18**, **P19**, and **P20** was noteworthy. Nonspecific interactions were evaluated by analyzing the response toward BSA, which was also spotted on the chip. Peptide correlation to BSA would indicate lack of specificity; however, no significant associations were detected over the matrix. Peptides like **P1**, **P2**, **P21**, and **P22** showed minimal correlation, explaining the lower immunogenic response.

Furthermore, Figure 4 also shows the correlation studies carried out in separate clusters to compare the signal recorded for the whole proteins and the corresponding peptide epitope related sequences. In this case, as expected, the cluster that showed the most significant peptide–protein correlation was the N group (NC-SARS2, NC-SARS, **P9**, **P10**, **P17**, **P18**, **P19**, and **P20**). Remarkably, all of the sequences of the N clusters showed significantly similar correlations. The high immunogenicity of the N structural protein^81^ could explain the excellent behavior observed for the related peptide epitopes, suggesting a potential for the possibility of using them on serological diagnostic devices, instead of the complete protein, which would allow the cost of such technology to be reduced even more.

With respect to the S1 subunit cluster, only **P12** showed a strong correlation with the recombinant parts of S1 evaluated as S1 trimer and S1 from SARS-2 and SARS viruses. This correlation highlights a comparable immunological performance between the evaluated linear sequence (T_553_ESNKKFLPFQQFGR) and the native structure of the S1 subunit. Previous reports have also postulated this exact sequence to be highly immunogenic describing, additionally, a strong linear dependence with virus neutralizing antibody titers which pointed to the neutralizing capacity of protein regions that are independent from the RBD region^82^ due to close proximity. On the other hand, no considerable peptide correlations toward the RBD portion could be recorded on these analyses. This may suggest that the three-dimensional geometry of the RBD could be fundamental for the recognition of neutralizing antibodies and further editions of the *Immuno*-μSARS2 chip should consider incorporating conformational peptides instead of linear sequences. This is in agreement with the results reported by Gattinger et al., who demonstrate that antibodies against RBD are detectable only with the folded version of the protein and not with the unfolded sequence, highlighting the importance of spatial epitope configuration.^80^ Nevertheless, at least for this application, the RBD epitope cannot be replaced for any of the evaluated peptide sequences.

### Clinical Validation of the *Immuno*-μSARS2 Chip as Tool to Assess Virus Exposure

**Figure 3:**
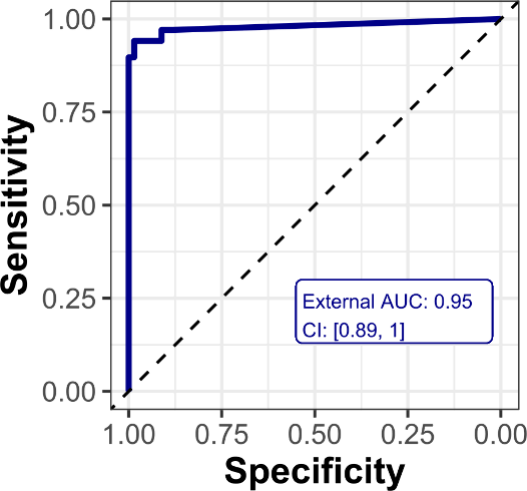
ROC curve of the Immuno-μSARS2 chip. The ROC curve with 0.95 AUC was obtained from a multivariate analysis of the hybrid peptide–protein matrix toward the discrimination of the RT-PCR+ group (*n* = 288) and control group (*n* = 96) IgG responses.

The clinical specificity and sensitivity of the chip were determined by analyzing 418 human serum samples from the three different cohorts, constituted by 312 RT-PCR+ samples and 97 prepandemic samples as negative control. The response from prepandemic samples was used to define the signal from nonspecific binding (see Figure 2), while RT-PCR+ samples showed a significant interaction with the epitopes in the matrix. Based on these results, the ROC curve was generated considering the interaction with the complete array matrix. As can be seen in Figure 3, a value of 0.95 area under the curve (AUC) was obtained, which correlated with 98% clinical specificity and 91% clinical sensitivity for the seropositive samples, which are encouraging results with respect to the potential implementation of the platform on prospective clinical or epidemiological studies.

**Figure 4:**
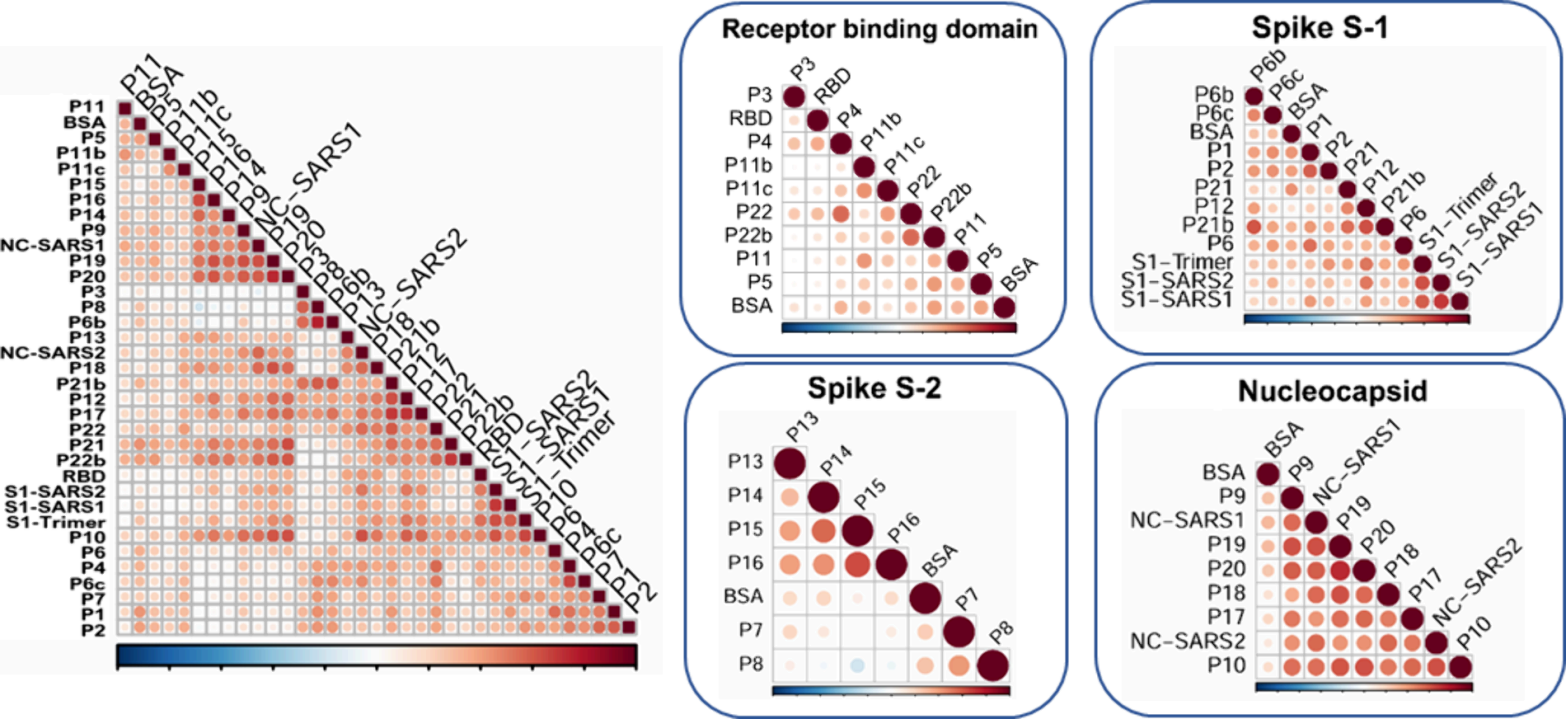
Pearson’s correlation coefficients represented according to (a) complete matrix of epitopes analyzed; (b) correlations based on structurally and conformationally related peptides and proteins.

### Studies on the Potential Prognostic Value of the *Immuno*-μSARS2 Chip

One of the main objectives of this research work was to assess a possible correlation between the serological signatures recorded and the clinical outcomes. For this purpose, RT PCR+ samples were classified according to the clinical history as (i) patients that required hospitalization (119), (ii) patients admitted to ICU (58), (iii) patients that were *exitus* (48), and (iv) patients with mild or asymptomatic disease (63). The IgG patterns of the first three groups were compared to those of the last one using machine learning techniques and classification models.

A limitation of this study pertains to the unavailability of precise dates indicating when patients transitioned to the three severity states, hindering the ability to accurately determine the lead time for predicting the severity. As a result, the predictions varied in terms of the number of days in advance, making it challenging to precisely ascertain. For future prospective studies, obtaining specific patient information, including admission dates, ICU dates, and date of death as well as the longitudinal study through time from the hospital would facilitate the calculation of lead times and enhance the accuracy of severity predictions. This approach would enable researchers to elucidate the temporal relationship between serological signatures and clinical outcomes, thus providing valuable insights into the predictive capabilities of serology in disease severity assessment.

The IgG immunological profiles recorded on each case appear to significantly predict the probability to require hospitalization, ICU admission, or *exitus* as it is shown in Table 2. A specific IgG signature could predict the fatal outcomes with 82% of accuracy (*P* = 0.0007, specificity of 84%, and sensitivity 80%). High sensitivity is associated with true positive results assuming that the immunological profile is exclusive for patients resulting in irreversible condition. For those with ICU admission, prediction could be achieved with an 80% accuracy (*P* = 0.001, specificity 82%, sensitivity of 78%), while for hospitalization, the accuracy was 73% accuracy (*P* = 0.008, specificity 63%, and sensitivity 83%).

**Table 2:** Severity Prediction Based on IgG Profile Coupled to Machine Learning Techniques

Clinical Outcome	Accuracy	*P* value^a^	Specificity	Sensitivity	Sd^a^
Hospitalization	73%	0.008^a^	63%	83%	6%
ICU admission	80%	0.001^a^	82%	78%	6%
*exitus*	82%	0.0007^a^	84%	80%	6%

a Statistically significant *P* < 0.05. Sd, standard deviation.

Relevant discussion arises behind the possibility to discriminate with the highest accuracy and statistical significance the three stages of severity compared with mild or asymptomatic signatures. The relevant correlation between humoral response mediated by IgG’s in patients that are more likely to undergo irreversible outcomes days before showing clinical manifestations. In this regard and according to our results, the clinical progression toward more severe outcomes directly influences the accuracy over classification method, considering the epitope matrix assessed, suggesting that an early imbalance during adaptive immune response development could be decisive for successful disease control. Nevertheless, additional indicators can be included in the microarray aiming to increase the predictive capacity of the platform such as, for instance, interleukins and other biomarkers from the innate immunological response.

## Conclusions

Theoretical modeling tools and available literature defining common immunodominant sequences have allowed selection of a panel of peptide epitopes from the S and N structural proteins of the virus in an attempt to select the most suitable candidates for profiling the complete immunological response of patients infected by the SARS-CoV2 virus and to correlate such profiles with the severity of the disease. A panel of 28 SARS-CoV-2 peptide sequences has been successfully synthesized, characterized by MS, and further bioconjugated to BSA. These bioconjugates plus additional epitopes (viral proteins and controls) have allowed the development of the *Immuno*-μSARS2 chip, a multiplexed fluorescent microarray to determine IgG and IgM titers, which can be manufactured with a high batch-to-batch reproducibility. Analyses performed with the *Immuno*-μSARS2 only require 10 μL of human serum samples and have been used in a high-throughput platform configuration, which allows the simultaneous analysis of 96 samples in just 90 min.

The initial evaluation of the chip was performed by analyzing 418 samples, delivering data which after multivariate analysis indicated that the *Immuno*-μSARS2 chip can provide results with a 98% specificity and 91% sensitivity toward RT-PCR+ classification, indicating remarkable analytical performance. Subsequently, the extended analyses of a total of 755 human serum samples has allowed the identification of peptides clusters derived from the S2 subunit and the N protein (**P10**, **P12**, and **P14**, **P15**, **P16** with **P17**, **P18**, **P19**, **P20**) with increased immunodominance in contrast with other regions evaluated. Pearson correlations were determined to explore common signal responses in the multiplexed matrix. In this regard, **P10** showed the best correlation with the structural protein and clinical predictive value while the rest of N derived peptides were also highly correlated with protein behavior. Simultaneously, **P12** was correlated to the S1 subunit, and based on literature reports, a neutralizing activity independent from RBD was described for this section. In addition, linear peptides derived from RBD showed weak response in contrast with the native portion, suggesting that conformational epitopes may be essential for NAb’s determination.

A distinctive immunological fingerprint was observed across RT-PCR+, RT-PCR–, and prepandemic samples evidencing a 20–25% of false negative results in RT-PCR– samples with positive serology. This platform allows for accurate differentiation of current and previous viral infection with high-throughput screening capabilities. In addition, the use of classification models in combination with machine learning techniques allowed the establishment of patient based serological signatures (mediated by IgG’s) to predict clinical outcomes. This resulted in specific antibody fingerprints predicting with 82% accuracy patients with fatal outcomes (*exitus*), with 80% accuracy those requiring ICU admission, and with 73% accuracy those hospitalized. These results demonstrate our initial hypothesis and point to the great potential of this diagnostic approach developed in this work to assess prognostic factors of SARS-CoV2 virus infection. The proposed approach could be extended to the diagnosis of other infectious diseases, which can be used in combination with other established technologies. The potential behind the integration of multiplexed diagnostic tools with biostatistical analysis directly influences the interpretation of the results, and this work demonstrates that multidisciplinary approaches are highly advantageous for future diagnostic applications.

## Supporting Information Available

Rational peptide design, peptide sequences synthesized for an array, synthesis of peptide epitopes 1–22, peptide BSA conjugate preparation, immunoreagents and spotting concentrations, slide derivatization protocol, fluorescent microarray development, studies on interday variability, studies on the variants of concern, interpretation of the immunoarray predictive value in RT-PCR negative human serum samples, and univariate analysis (PDF)
