# Supplementary material for: Immuno-μSARS2 Chip: A Peptide-Based Microarray to Assess COVID-19 Prognosis Based on Immunological Fingerprints
Source: ACS Pharmacol Transl Sci. 2025 Feb 21;8(3):871–84. doi: 10.1021/acsptsci.4c00727 (PMC11915183; doi:10.1021/acsptsci.4c00727)
Supplement: Supplementary file 1 — pt4c00727_si_001.pdf [file pt4c00727_si_001.pdf]

Supporting Information

Immuno-µSARS2 chip: a peptide-based microarray to assess COVID-19 prognosis based on immunological fingerprints.

Julian Guercetti[1,3], Marc Alorda[1,3,9,10], Luciano Sappia[1,3], Roger Galve[1,3], Macarena Duran-Corbera[2,3], Daniel Pulido[2,3], Ginevra Berardi[2,3], Miriam Royo[2,3],Alicia Lacoma[4,5], José Muñoz[6], Eduardo Padilla[6], Silvia Castañeda[7], Elena Sendra[7], Juan P. Horcajada[7,8], Agustín Gutierrez-Galvez[9,10], Santiago Marco[9,10], J.-Pablo Salvador[1,3] and M.-Pilar Marco[1,3]

1. Nanobiotechnology for Diagnostics Group. Instituto de Química Avanzada de Cataluña, IQAC-CSIC, C/ Jordi Girona 18-26, 08034 Barcelona.
2. Multivalent Systems for Nanomedicine (MS4N). Instituto de Química Avanzada de Cataluña, IQAC-CSIC, C/ Jordi Girona 18-26, 08034 Barcelona
3. CIBER de Bioingeniería, Biomateriales y Nanomedicina (CIBER-BBN), Spain
4. Hospital Universitari Germans Trias i Pujol, Badalona, Spain, Institut Germans Trias i Pujol, Badalona, Spain
5. CIBER Enfermedades Respiratorias (CIBERES), Badalona, Spain
6. Servicio de Microbiología del Laboratorio de Referencia de Catalunya
7. Servicio de Enfermedades Infecciosas del Hospital del Mar de Barcelona, COVID-MAR group
8. CIBER de Enfermedades Infecciosas (CIBERINFEC), Instituto de Salud Carlos III, Madrid, Spain
9. Institute for Bioengineering of Catalonia (IBEC), The Barcelona Institute of Science and Technology, Baldiri Reixac 10-12, 08028, Barcelona, Spain

10 Department of Electronics and Biomedical Engineering, University of Barcelona, Marti i Franqués 1-11, 08028 Barcelona, Spain

*Corresponding Author:
Dr. J.-Pablo Salvador
Nanobiotechnology for diagnostics group
CIBER-BBN/IQAC-CSIC
Jordi Girona, 18-26
08034-Barcelona
Spain
Phone: +34 93 4006100, Ext 437892
E-mail: jpablo.salvador@iqac.csic.es

Experimental section

Rational peptide design was implemented towards the selection of discrete immunogenic sequences synthesized

Rational peptide design

In order to predict B-cell epitopes directly from the protein sequence, the BepiPred-2.0 software was employed as supporting platform towards the rational selection of peptide candidates. This server uses a Random Forest algorithm that has been trained on both epitopes and non-epitope amino acids obtained from crystal structures, taking into account a helix (pink), b sheet (blue) and random coil (Orange) configurations across the protein structure, as well as accessible and non-accessible regions. The FASTA sequences included in the analysis were obtained from the Uniprot database for Spike glycoproteins (P0DTC2) and nucleocapsid (P0DTC9), defining an epitope threshold of 0.6 to detect restrictive predictions. Simultaneously, each peptide candidate was represented across the sequence to provide direct identification and structural distribution. All peptides were synthesized as linear sequences.

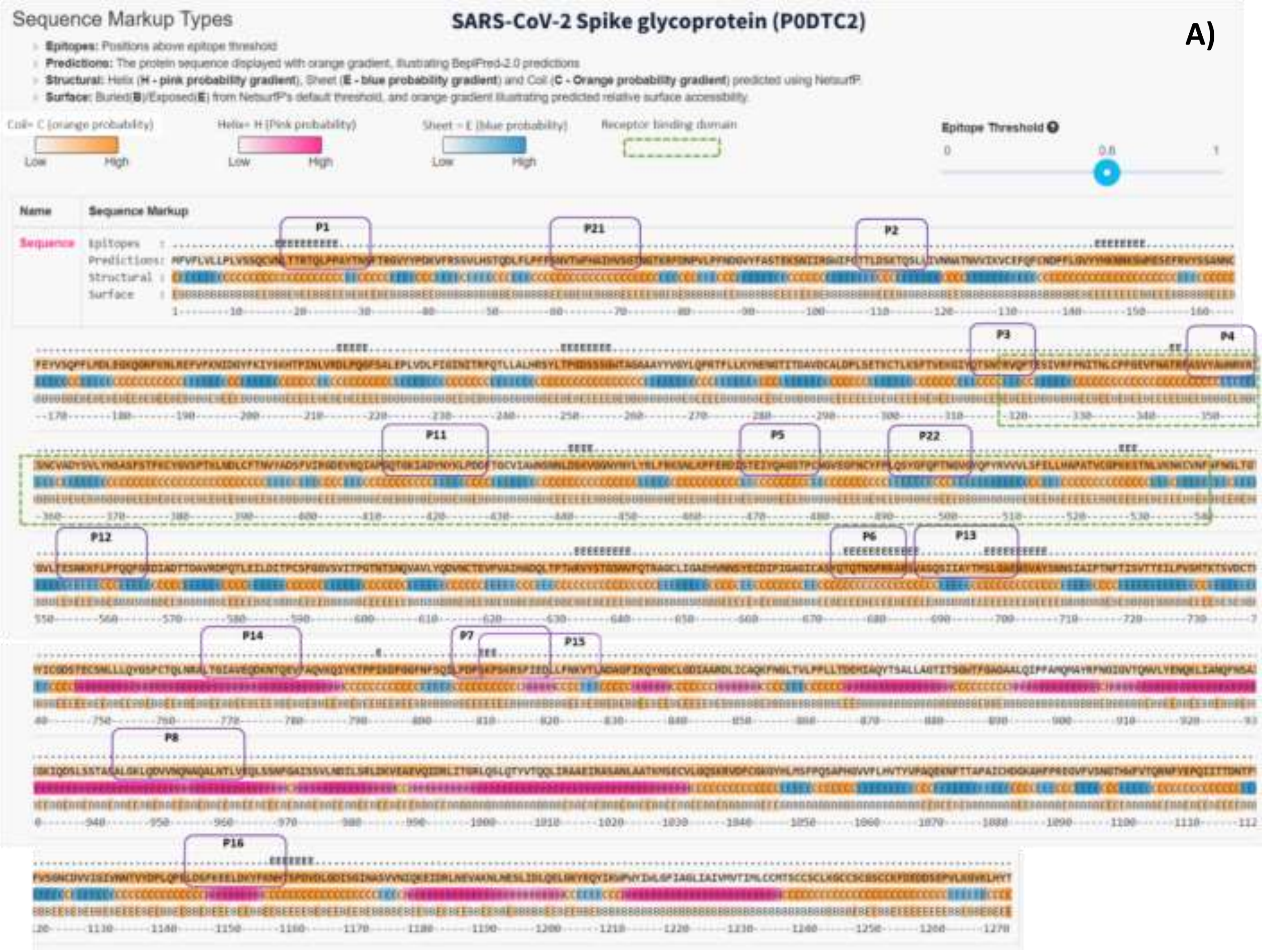
Sequence Markup Types
SARS-CoV-2 Spike glycoprotein (P0DTC2)
A)
Epitopes: Positions above epitope threshold
Predictions: The protein sequence displayed with orange gradient, illustrating BepiPred-2.0 predictions
Structural: Helix (H - pink probability gradient), Sheet (E - blue probability gradient) and Coil (C - Orange probability gradient) predicted using NetsurfP.
Surface: Buried(B)/Exposed(E) from NetsurfP's default threshold, and orange gradient illustrating predicted relative surface accessibility.
Coil= C (orange probability)
Helix= H (Pink probability)
Sheet = E (blue probability)
Receptor binding domain
Low
High
Epitope Threshold
0
0.8
1
Name
Sequence Markup
Sequence
Epitopes
Predictions
Structural
Surface
P1
P21
P2
P3
P4
P11
P5
P22
P12
P6
P13
P14
P7
P15
P8
P16

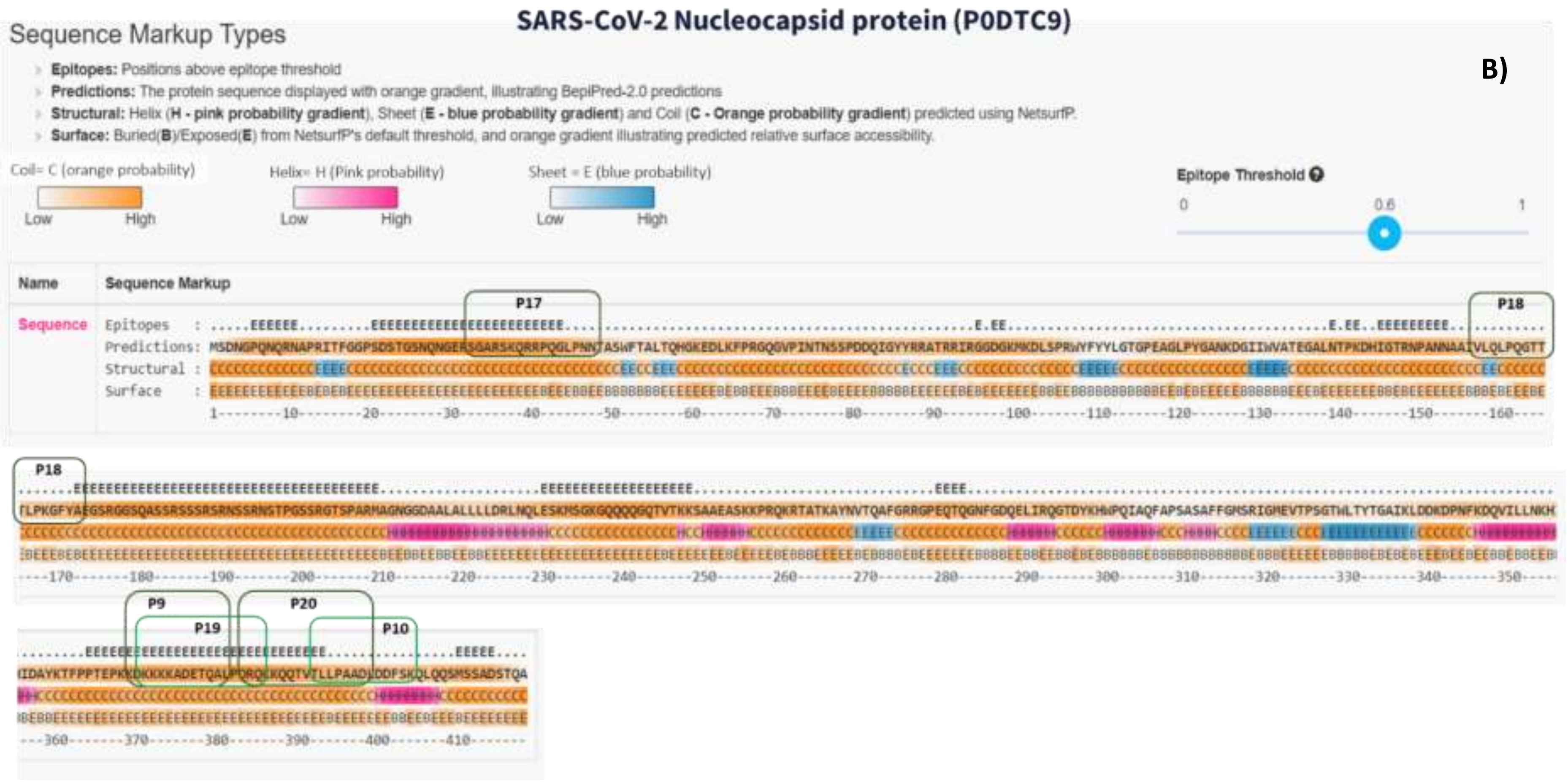

Figure S1. Results from B cell epitope prediction analysis. The open access software BepiPred 2.0 was utilized to predict potential B cell epitopes based on the structure of S (above) and N (below) protein sequences extracted from protein data bank (PDB). The software estimates probable epitopes position (with a threshold of 0.6) assigned with the letter E over the sequence. Also, in the structural row, highlight in orange the probability of random coil conformation, in pink the α-helix conformation and in blue the β-sheet structures considering the amino acids involved. Additionally, in the row indicated as surface, the letter

E corresponds to exposed amino acids while the letter B refers to buried amino acids. Moreover, the final peptide sequence selected is indicated on the protein sequences with their corresponding numbers. A) S protein sequence is expressed at amino acid resolution indicating target regions for each peptide. B) N protein sequence was analysed and the peptides selected are represented.

In addition, several factors were considered as selection criteria for the final peptides like antigenicity, searching for regions with high antigenic scores, and also the evaluation of hydrophobic/hydrophilic balance of the proteins, considering that epitopes exposed to the surface evidence a characteristic hydration ratio. Moreover, the length of the sequence was typically defined towards peptides between 12 and 15 mers, with a preference for non-conserved regions, which are generally more immunogenic than conserved ones. For example, regions containing helix-loop-helix and β-sheet structural groups are commonly found in several proteins and therefore should be avoided during this type of selection. In terms of accessibility, exposed loops are more prone to interact with antibodies as conformational epitopes becoming interesting targets.

Table S1: Peptide sequences synthesized for array

The final peptide selection is presented in the following table, which shows the target viral sequence (of interest),the actual sequence synthesized, the protein region correspondence, the peptide purity after synthesis and the peptide density estimated over the BSA bioconjugates.

Peptide	Target Viral sequence	Synthesized Sequence	Protein region	Purity (%)	BSA-peptide (δ) density
**Peptide 1**	L_{18}TTRTQLPPAYTNS	Ac-CLTTRTQLPPAYTNS-NH_2	S1-Spike	98	5
**Peptide 2**	L_{110}DSKTQ	Ac-CTTLDSKTQSLL-NH_2	S1-Spike	99	5
**Peptide 3**	Q_{314}TSNFRVQPT	Ac-CQTSNFRVQPT-NH_2	S1-Spike (RBD)	99	5
**Peptide 4**	A_{352}WNRKR	Ac-CASVYAWNRKRISN-NH_2	S1-Spike (RBD)	99	6
**Peptide 5**	S_{469}TEIYQAGSTPC	Ac-TEIYQAGSTPC-NH_2	S1-Spike (RBD)	99	6
**Peptide 6**	$\mathbf{Y_{674}}$QTQTNSPRRAR	Ac-CYQTQTNSPRRAR-NH_2	S1-Spike	99	5
**Peptide 7**	L_{806}PDPSKPSKRSFIED	Ac-CLPDPSKPSKRSFIED-NH_2	S2-Spike	99	5
**Peptide 8**	A_{944}LGKLQDVVNQNAQALNTLV	Ac-CALGKLQDVVNQNAQALNTLV-NH_2	S2-Spike	92	5
**Peptide 9**	K_{370}DKKKKADETQAL	Ac-CKDKKKKADETQAL-NH_2	Nucleocapsid	98	4
**Peptide 10**	T_{393}LLPAADLDDFSK	Ac-CTLLPAADLDDFSK-NH_2	Nucleocapsid	95	8
**Peptide 11**	G_{413}QTGKIADYNYKLPDD	Ac-CGQTGKIADYNYKLPDD-NH_2	S1-Spike (RBD)	94	6
**Peptide 12**	T_{553}ESNKKFLPFQQFGR	Ac-CTESNKKFLPFQQFGR-NH_2	S1-Spike	92	7
**Peptide 13**	V_{687}ASQSIIAYTMSLGAE	Ac-VASQSIIAYTMSLGAEC-NH_2	S2-Spike	91	7
**Peptide 14**	L_{767}TGIAVEQDKNTQEV	Ac-CLTGIAVEQDKNTQEV-NH_2	S2-Spike	98	5
**Peptide 15**	S_{813}KRSFIEDLLFNKVTL	Ac-CSKRSFIEDLLFNKVTL-NH_2	S2-Spike	98	6
**Peptide 16**	L_{1145}DSFKEELDKYFKNH	Ac-CLDSFKEELDKYFKNH-NH_2	S2-Spike	97	6
**Peptide 17**	S_{33}GARSKQRRPQGLPNN	Ac-CSGARSKQRRPQGLPNN-NH_2	Nucleocapsid	91	5
**Peptide 18**	V_{158}LQLPQGTTLPKGFYA	Ac-CVLQLPQGTTLPKGFYA-NH_2	Nucleocapsid	94	6
**Peptide 19**	D_{371}KKKKADETQALPQRQ	Ac-CDKKKKADETQALPQRQ-NH_2	Nucleocapsid	92	5
**Peptide 20**	Q_{384}RQKKQQTVTLLPAAD	Ac-CQRQKKQQTVTLLPAAD-NH_2	Nucleocapsid	98	4
**Peptide 6b**	Y_{674}QTQTNSHRRAR	Ac- CYQTQTNSHRRAR -NH_2	S1-Spike	95	6
**Peptide 6c**	Y_{674}QTQTKSHRRAR	Ac-CYQTQTKSHRRAR-NH_2	S1-Spike	96	4

**Peptide 11b**	G_{413}QTGNIADYNYKLPDD	Ac-CGQTGNIADYNYKLPDD-NH_2	S1-Spike (RBD)	>99	7
**Peptide 11c**	G_{413}QTGTIADYNYKLPDD	Ac-CGQTGTIADYNYKLPDD-NH_2	S1-Spike (RBD)	>99	7
**Peptide 21**	S_{60}NVTWFHAIHVSGT	Ac-CSNVTWFHAIHVSGT-NH_2	S1-Spike	>99	7
**Peptide 21b**	S_{60}NVTWFHAISGT	Ac-CSNVTWFHAISGT-NH_2	S1-Spike	99	10
**Peptide 22**	LQSYGFQPTN_{501}GVG	Ac-CLQSYGFQPTNGVG-NH_2	S1-Spike (RBD)	98	8
**Peptide 22b**	LQSYGFQPTY_{501}GVG	Ac-CLQSYGFQPTYGVG-NH_2	S1-Spike (RBD)	96	10

- **Synthesis of peptide epitopes 1-10**

The first generation of peptide epitopes (peptides **1-10**) were synthesized manually following the standard Fmoc/*t*Bu solid-phase strategy and using Fmoc-Rink Amide CM (0.50 mmol/g) as the polymeric support. The resin was first conditioned with dimethylformamide (DMF, 3 x 1 min), dichloromethane (DCM, 3 x 1 min) and DMF (3 x 1 min). The Fmoc group was then removed by treating the resin with a solution of 20% piperidine in DMF (2 x 5 min) and the resin was washed with DMF and DCM. The C-terminal residue was incorporated by adding the Fmoc protected amino acid (3 equiv) together with N,N'-diisopropylcarbodiimide (DIC, 3 equiv) and ethyl cyanohydroxyiminoacetate (Oxyma, 3 equiv) in DMF for 1 h at rt. The peptide sequence was elongated by cycles of Fmoc removal and coupling of the following amino acid. All the synthesized peptides were acetylated at the N-terminal end of the peptide. Acetylation was achieved by treating the peptidyl-resin with acetic anhydride (Ac_2O, 10 equiv) and N,N-diisopropylethylamine (DIEA, 20 equiv) in DCM for 30 min at rt and followed by washing the peptidyl-resin with DCM (3 x 1 min), DMF (3 x 1 min) and DCM (3 x 1 min).

Cleavage of the peptide from the resin was achieved by treating the peptidyl-resin with a mixture of trifluoroacetic acid-triisopropylsilane-water (TFA:TIS:H_2O, 94:3:3, v/v/v) for 2.5 h. The solution was filtered and the peptide was precipitated by adding of the TFA solution to cold diethylether (Et_2O). The precipitate was centrifuged, and the supernatant was discarded. The peptide was washed twice with Et_2O, dissolved in water-acetonitrile and lyophilized.

All the peptides were purified using by a semi-preparative high-performance liquid chromatography (HPLC). Crudes and purified peptides were analyzed by HPLC using a XBridge BEH C_{18} column (4.6 x 100 mm, 3.5 µm) and a linear gradient of acetonitrile (0.036% TFA) in water (0.045% TFA) in 8 min and a flow rate of 1.0 ml min^{-1} and HPLC-MS using a XSelect C_{18} column (4.6 x 50 mm, 3.5 µm) and a linear gradient of 5-100% of acetonitrile (0.07% HCOOH) in water (0.1% HCOOH) in 3.5 min and a flow rate of 1.6 ml min^{-1}. The purity was determined by HPLC (UV).

- **Synthesis of peptide epitopes 11-20, 6b-c, 11b-c, 21, 21b, 22 and 22b**

The second generation of peptide epitopes (peptides **11-20**) and the epitopes resulting from the emerging variants of SARS-CoV-2 (peptides **6b-c**, **11b-c**, **21**, **21b**, **22** and **22b**) were synthesized following the standard Fmoc/*t*Bu solid-phase strategy in an automated microwave-assisted peptide synthesizer (Liberty Blue, CEM). Fmoc-Rink Amide ProTide (0.56 mmol/g) was used as the polymeric support for the syntheses. The Fmoc removal and coupling reactions were carried out according to standard protocols. The coupling reactions were performed using a solution 0.5 M of DIC in DMF as coupling reagent and 1.0 M of Oxyma in DMF as additive. All the synthesized peptides were acetylated at the N-terminal end of the peptide as described above.

Cleavage of the peptide from the resin was achieved by treating the peptidyl-resin with a mixture of TFA-TIS-2,2'-(ethylenedioxy)diethanethiol (ODT) (95:2.5:2.5, v/v/v) for 2.5 h. The solution was filtered and the peptide was precipitated by adding the TFA solution to cold Et_2O, centrifuged, and the supernatant was discarded. The peptide was washed twice with Et_2O, dissolved in water-acetonitrile and lyophilized.

The peptides were purified using a semi-preparative HPLC and analyzed with the chromatographic conditions described above.

Peptide BSA conjugates

The previous table summarizes the peptide densities obtained per molecule of BSA conjugated. Characterization was made by MALDI-TOF. Each peptide was resuspended to a 1 mg/mL stock in PBS 10 mM and then separated in aliquots of 100 µL stored at -20ºC until use. The spotting bioconjugate concentration was set at 0.5 mg mL^{-1} in printing buffer.

Peptide BSA conjugation protocol: A BSA dilution is prepared at 10 $mgmL^{-1}$ in PBS buffer (30 mg/3 mL PBS 10 mM). Then, 2,08 mg of N-SMP were diluted in 100 µL DMF (Dimethylformamide). The N-SMP solution was added dropwise over the BSA solution and left for 1 hour at rt and overnight at 4ºC under stirring. The following day, N-SMP conjugated BSA was purified by ÄKTA prime plus (GE Healthcare) at a 1 mL/min in PBS buffer and final concentration was estimated through Bradford Test.

The molar ratio of BSA-linker and peptides was established at 1:15. Then, peptides were reconstituted in ultrapure water (MilliQ H_2O) and hydrophobic peptides were resuspended in a mixture of ACN:H_2O. Simultaneously, 100 µL of tris(2-carboxyethyl) phosphine (TCEP) solution at 1.3mg.mL^{-1} were added over resuspended peptides for 10 min at 37ºC to avoid disulphide bound formation. Then, peptide and TCEP solution was added dropwise over BSA-linker solution and left 1 h under stirring at rt andovernight at 4ºC.

Finally, peptide conjugates were purified by dialysis against 0.5 mM PBS (4 × 5 L) and MilliQ water (1 × 5 L) and stored freeze dried at -40 °C. Unless otherwise indicated, working aliquots were stored at 4 °C in PBS at 1 mg mL^{-1}.

Bioconjugates characterization was performed by MALDI-TOF, were 2 µL of the freshly prepared matrix (trans-3,5-dimethoxy-4-hydroxycinnamic acid, 10 mg mL^{-1} in ACN/H2O 70:30, 0.1% HCOOH) was mixed with 2 µL of a solution of the native protein or the bioconjugates (10 mg mL^{-1} in the same solvent). Peptide densities were estimated according to the following equation: [MW (conjugate)-MW(protein)]/MW(peptide).

Immunoreagents and spotting concentrations

Epitopes concentrations were optimized with a serum provided from commercial source to minimize background noise and non-specific adsorptions. All dilutions were prepared in printing buffer and then subsequently dispensed over derivatized glass slides as explained.

Table S2-Protein immobilization conditions over microarray matrix

Protein epitope	Concentration (µg mL^{-1})	Commercial source	Protein epitope	Concentration (µg mL^{-1})	Commercial source
S1-SARS2	12.5	Acro Biosystems (S1NC52H3)	NC-SARS	6.25	The Native Antigen Company
S1-SARS1	12.5	The Native Antigen Company	Peptide-BSA	500	In-house
S1-Trimer-SARS2	64	Protein Production Platform*	IgG from Human serum	50	Sigma-Aldrich (I4506)
RBD$_2$	20	RayBiotech (HEK)	IgM from Human serum	5	Sigma-Aldrich (I8260)
BSA	500	Sigma-Aldrich	Rabbit PAb to Human IgG (TRITC)	5	ABCAM (ab6756)
NC-SARS2	6.25	Acro Biosystems	Rabbit PAb to Human IgM (Alexa fluor 647)	5	ABCAM (ab150191)

* S1-Trimer-SARS2 was produced in the protein production platform according to the following protein sequence extracted from: “Microneedle array delivered recombinant coronavirus vaccines: Immunogenicity and rapid translational development. Kim, Eun et al. eBioMedicine, Volume 55, 102743”

QC Antisera: In house generated rabbit antisera were utilized as QC reagents to characterize the signal reproducibility after each printing session. Female New Zeeland Rabbits were immunized with S1 recombinant proteins to produce As410 and with N recombinant proteins to obtain As414, specific for each target protein.

Slide derivatization protocol

This protocol was performed over of 20 glass slides (Corning, 75x25mm) simultaneously. To start they were washed with soap and ultrapure water to then be immersed in Piranha solution (H2SO4:H2O2, 70:30, v/v) for 30 min. Then were rinsed 6 times with ultrapure Water and activated with 10 % NaOH for 60 min. After that, 3 washes with Mili-Q-water and 3 with 70 % EtOH were required previously to the addition of GPTMS solution. For this purpose, 250 µL of GPTMS in EtOH abs (242.5 µL absolut EtOH + 1.68 µL glacial acetic acid + 5.83 µL of GPTMS) were incubated with the slides for 3 h at rt. Finally, the slides were rinsed with 96 % EtOH 3 times, then sonicated for 20 minutes in absolute ethanol and dried with N_2 flow and stored them in desiccator chamber for 1 month in vacuum.

Fluorescent microarray

Microarray measurements.

The spots were measured by deducting the mean fluorescence background intensity to the mean of fluorescence foreground intensity using a two-colour microarray scanner InnoScan 710, (Innopsys, Carbonne, France) at different wavelengths simultaneously (555 nm for IgG

determination and 647 nm for IgM detection). Mapix analysis software *v 7.4.0* (Innopsys Inc., Carbonne, France) was employed for slide signal acquisition under following conditions: 555 nm channel (green, IgG) Gain=3, Power Low 5mW and 647 channel (red, IgM) Gain=10, Power Low 5mW. Excel tables were generated and predesigned templates utilized to assign intensity values to the respective epitopes calculating the mean fluorescence of 3 replicate spots + 2 SD. Then, GraphPad Prism V 7.0 (GraphPad Software Inc., San Diego, CA, USA) was used to generate heatmaps showing the intensity of the group of slides per analysis in every sample and for every epitope.

Spot optimization parameters

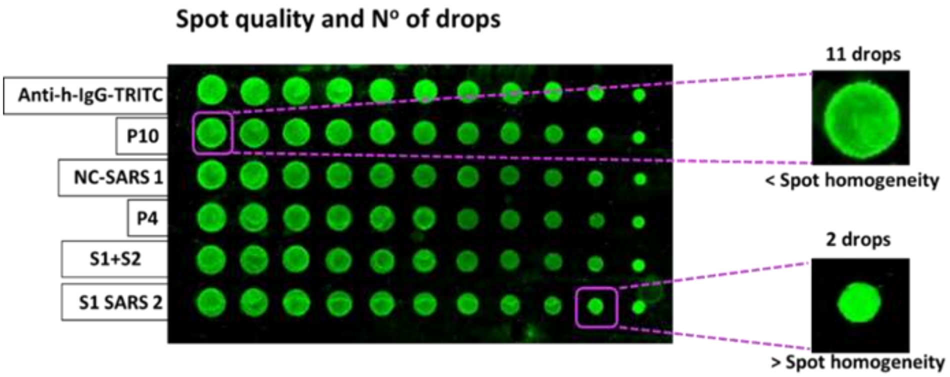

Figure S2 Micrography of a single sub-array containing the epitopes evaluated with different number of drops per spot from 1 to 11. Each epitope solution was printed on the same row varying the number of drops to compare spot homogeneity after incubation with a mixture of As410 (positive control for S) and As414 (positive control for N). The images were acquired using a microarray scanner with a laser at excitation wavelength of 532 nm.

Smaller numbers of drops per spot evidenced increased consistency within epitopes leading to lower inter-spot variability compared to higher amounts of drops. In addition, the signal distribution in spots with less than 2 drops was considerably more homogenous and higher fluorescence intensity was recorded on peptide-BSA conjugates. Taking this into consideration, chip's printing was defined in two drops per spot to generate smaller arrays limited by the ArrayIt® gasket dimensions.

Microarray spotting schema

Immuno-µSARS2 chip 2.0

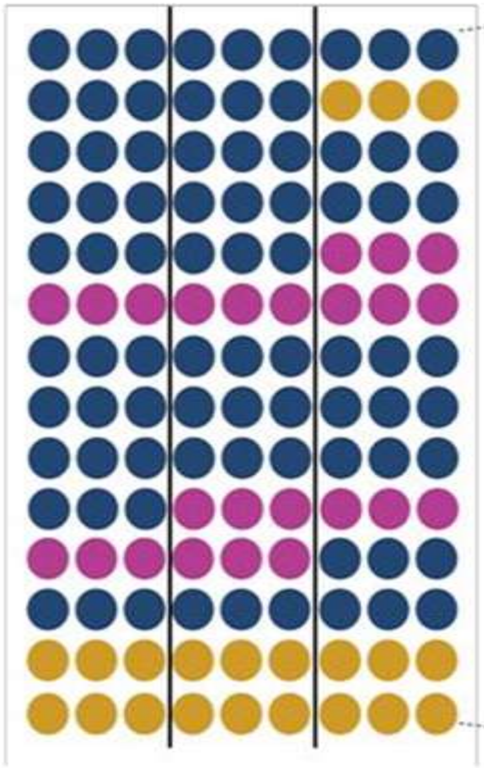

S1-SARS2	S1-SARS	S1 Trimer
S1+S2	RBD	BSA
P1	P2	P3
P4	P5	P6
P7	P8	NC-SARS2
NC-SARS	P9	P10
P6b	P6c	P11
P11b	P11c	P12
P13	P14	P15
P16	P17	P18
P19	P20	P21
P21b	P22	P22b
hIgG	hIgM	A_hIgG
A-hIgM	S1-SARS2	NC-SARS2

*Figura S3***.** *Immunoarray 2.0. Final epitope distribution of updated version of the microarray containing 35 different epitopes in triplicate spots to assess IgG/IgM mediated response after SARS CoV-2 infection including internal controls.*

Serum Samples:

All human serum samples were obtained with the corresponding ethical committees' approval established in Spain (Law 14/2007, 3rd of July, of Biomedical Research) with the consent of enrolled patients for research purposes. As received, working aliquots were prepared to avoid defrizzing cycles and the samples were stored at -80°C.

Inter-day variability:

Inter-day and batch to batch chip variability was evaluated making use of As410 (Anti-S) and As414 (Anti-N) at the three concentration (100, 50 and 25 µg mL^{-1}). The following graph S3, shows the results obtained with 100 µg mL^{-1} as representative example. For this experiment, the fluorescence response from single chips printed in four slides generated in separate batches and measured in different days was compared. Each microarray chip contained three replicate spots of each peptide-BSA conjugate and recombinant protein including the SD in each column and the CV between days was below 20% evidencing the reproducibility of chip's printing process.

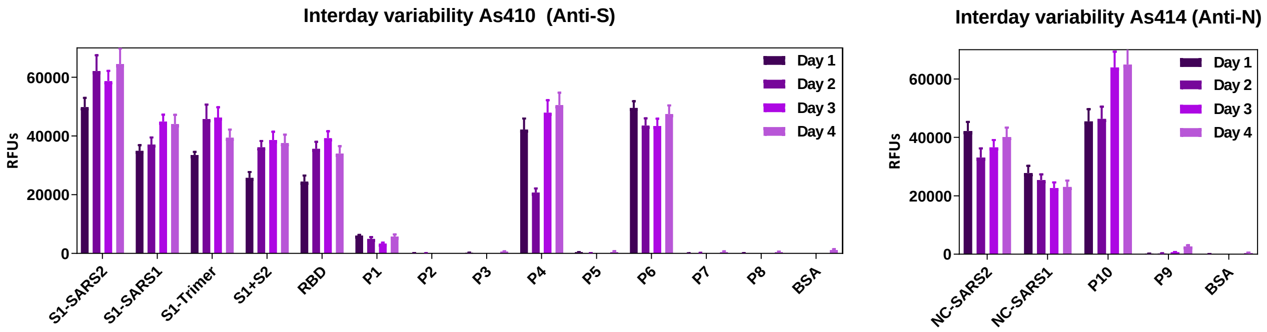

Figure S4. Inter-day variability using As410 (Anti-S) and As414 (Anti-N). Each bar shows the average and standard deviation of the fluorescence intensity recorded for each epitope printed in three replicate spots on each microarray chip (SD) and processed in a single day using the As410 and As414 at 100 µg mL^{-1}. Experiments were carried out in four different days. Each day an independent slide produced in different batches was used. The CV was below 20 %.

Variants of concern

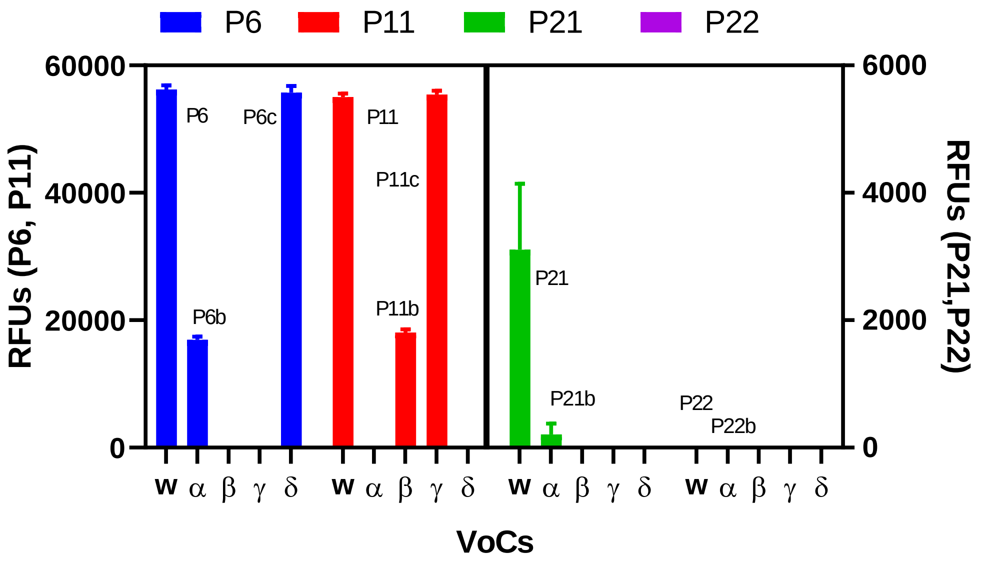

Figure S5. Immunological response using As410 (anti S) against P6, P11, P21 and P22 and their corresponding variant of concern (VoC) peptides sequences. P6$_{Ø,b,c}$-BSA, P11 $_{Ø,b,c}$-BSA, P21 $_{Ø,b}$-BSA and P22$_{Ø,b,c}$-BSA were immobilized at 0.5 mg·mL^{-1} and the concentration of As410 used for P6 and P11 was 5 µg·mL^{-1} and for P21 and P22 was 100 µg·mL^{-1}. The SD showed correspond to N=4

Interpretation of the immunoarray predictive value in RT-PCR negative human serum samples.

The interpretation of the results from the analyses of 288 classified as RT-PCR- had to consider three possible scenarios A) lack of present and past infection with both, RT-PCR and *Immuno-*µSARS2 chip providing negative results (as prepandemic samples) ; B) initial phase of infection where antibody titters and viral loads are non-detectable, therefore, negative results also with

both techniques; and C) late phase of infection with low or no viral load, but positive IgG response. This last scenario would be the one most commonly found when using the present platform for epidemiological studies, long term immune response assessment and investigation of the immunity induced by vaccines as well as.

The analysis of the samples from patients diagnosed to be RT-PCR- with the *Immuno*-µSARS2 chip, showed a 20-25 % rate of positive results in respect to the immunological response against the viral epitopes evaluated. Bearing in mind that these samples were collected during the initial stage of the pandemic (April), there exists the possibility that these patients could have been erroneously diagnosed at that moment, with a C scenario in which the low viral load would have led to negative results. Hence, it should be noticed that at that time, there was important constraints regarding access to the necessary reagents and standardized techniques. Moreover, the clinical knowledge of the disease was also poor which led to several misinterpretations in the precise identification of viral infection.

Univariate analysis:

Across the ***Univariate Model***, epitopes correlation was established and then the accuracy was estimated through ROC curves generation with their corresponding AUC values providing sensitivity and specificity for each component of the matrix classifying pre-pandemic vs RT-PCR+ patients. The correlation of the epitopes was calculated applying Pearson correlation coefficient with the Corrplot R package. The statistical significance and confidence interval of the ROC curves and AUC values was estimated using a binomial test. P-values for statistical analysis were adjusted using Benjamini – Hochberg correction. Analysis of significance and confidence interval for classification rate were obtained applying Wilcoxon test and bootstrap respectively.

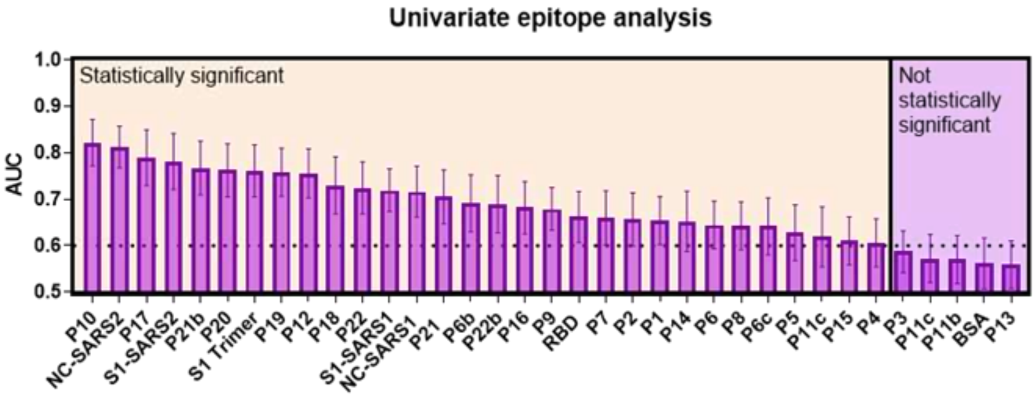

Figure S6. Individual epitope classification capacity. From the complete matrix utilized, a univariate analysis was conducted estimate the AUC value for each epitope discriminating RT-PCR+ samples from pre-pandemic samples. In this case, epitopes with AUC and Classification rate above 0.6 were statistically significant predictors.

In this regard**, P10** showed the higher AUC value indicating accurate predictive capacity even over the NC-SARS2 and S1-SARS2 recombinant proteins. Interestingly, 30 of the 35 epitopes assessed found to be significant individual classifiers. Remarkably, BSA (included as control) didn’t showed significant correlation, validating the specificity of the peptide sequences selected for the analysis.
